# Molecular and Biochemical Analysis of Two Rice Flavonoid 3’-Hydroxylase to Evaluate Their Roles in Flavonoid Biosynthesis in Rice Grain

**DOI:** 10.3390/ijms17091549

**Published:** 2016-09-13

**Authors:** Sangkyu Park, Min Ji Choi, Jong Yeol Lee, Jae Kwang Kim, Sun-Hwa Ha, Sun-Hyung Lim

**Affiliations:** 1 National Institute of Agricultural Science, Rural Development Administration, JeonJu 54874, Korea; psk2779@korea.kr (S.P.); nancmj90@naver.com (M.J.C.); jy0820@korea.kr (J.Y.L.); 2 Division of Life Sciences, College of Life Sciences and Bioengineering, Incheon National University, Incheon 22012, Korea; kjkpj@inu.ac.kr; 3 Department of Genetic Engineering and Graduate School of Biotechnology, Kyung Hee University, Yongin 17104, Korea; sunhwa@khu.ac.kr

**Keywords:** anthocyanins, flavonoid 3′-hydroxylase, flavonoids, leucocyanidins, pigmented rice, proanthocyanidins

## Abstract

Anthocyanins and proanthocyanidins, the major flavonoids in black and red rice grains, respectively, are mainly derived from 3′,4′-dihydroxylated leucocyanidin. 3′-Hydroxylation of flavonoids in rice is catalyzed by flavonoid 3′-hydroxylase (F3′H: EC 1.14.13.21). We isolated cDNA clones of the two rice *F3′H* genes (*CYP75B3* and *CYP75B4*) from Korean varieties of white, black, and red rice. Sequence analysis revealed allelic variants of each gene containing one or two amino acid substitutions. Heterologous expression in yeast demonstrated that CYP75B3 preferred kaempferol to other substrates, and had a low preference for dihydrokaempferol. CYP75B4 exhibited a higher preference for apigenin than for other substrates. CYP75B3 from black rice showed an approximately two-fold increase in catalytic efficiencies for naringenin and dihydrokaempferol compared to CYP75B3s from white and red rice. The F3′H activity of CYP75B3 was much higher than that of CYP75B4. Gene expression analysis showed that *CYP75B3*, *CYP75B4*, and most other flavonoid pathway genes were predominantly expressed in the developing seeds of black rice, but not in those of white and red rice, which is consistent with the pigmentation patterns of the seeds. The expression levels of *CYP75B4* were relatively higher than those of *CYP75B3* in the developing seeds, leaves, and roots of white rice.

## 1. Introduction

**Figure 1:**
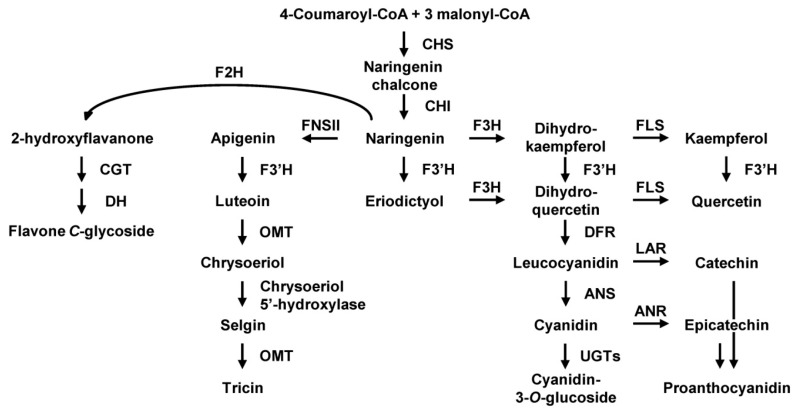
Proposed flavonoid biosynthesis pathway in rice grain. The abbreviations of enzyme names are as follows: CHS, chalcone synthase; CHI, chalcone isomerase; F3H, flavanone 3-hydroxylase; FLS, flavonol synthase; F3′H, flavonoid 3’-hydroxylase; DFR, dihydroflavonol 4-reductase; ANS, anthocyanidin synthase; UGT, UDP-glucosyl transferase; LAR, leucoanthocyanidin reductase; ANR, anthocyanidin reductase; FNSII, flavone synthase II; OMT, *O*-methyltransferase; F2H, flavanone 2-hydroxylase; CGT, *C*-glucosyl transferase; and DH, dehydratase.

Rice is a staple food in many Asian countries. Although white rice is most commonly consumed, pigmented rice is also used in Asian diets. Numerous lines of evidence suggest that pigmented rice has important biological activities, such as antioxidant [1,2,3,4], anti-tumor [5,6,7], anti-allergic [8,9], and neuro-protective activities [10]. Pigmented rice grains contain large amounts of flavonoids. The major flavonoids in black rice grains are anthocyanins, which are mainly composed of cyanidin-3-*O*-glucoside, and peonidin-3-*O*-glucoside, and in red rice grains are proanthocyanidins and flavan-3-ols oligomers, which have catechin as the main extension unit [11,12,13,14,15]. Additionally, small quantities of aglycones and glycosides of flavanones, flavones, dihydroflavonols, and flavonols are also present in black and red rice grains [13,15,16,17]. Flavonoids are barely detected in white rice grains, except for small quantities of tricin (3′,5′-dimethoxylated flavone) [18,19]. Tricin, like anthocyanins and proanthocyanidins, functions as a strong antioxidant in rice plants [20], and tricin derivatives were reported to be incorporated into rice lignin [21], thereby functioning in biotic and abiotic stress protection and plant growth. The flavonoid biosynthesis pathway in rice has been suggested by several studies, which identified the genes and enzymes involved in the pathway (Figure 1). In the first committed step, the activity of chalcone synthase (CHS) leads to the formation of chalcone, which is converted to naringenin by the action of chalcone isomerase (CHI). Naringenin is then used as a universal substrate for the biosynthesis of various flavonoids. Anthocyanin and proanthocyanidin biosynthesis both include steps that are catalyzed by flavanone 3-hydroxylase (F3H) and dihydroflavonol 4-reductase (DFR). Anthocyanidin is synthesized by anthocyanidin synthase (ANS), whereas proanthocyanidin is synthesized by leucoanthocyanidin reductase (LAR) during late steps of the pathway. F3′H, which catalyze B-ring hydroxylation of flavonoids, adds diversity to the composition of flavonoids in rice grains. Tricin is synthesized through a different pathway in which apigenin formed by the action of flavone synthase II (FNSII) is utilized. Flavanone 2-hydroxylase (F2H) converts naringenin to 2-hydroxyflavanone in the *C*-glycosylflavone biosynthesis pathway (Figure 1).

Most anthocyanins and proanthocyanidins accumulated in pigmented rice grains are commonly derived from 3′4′-dihydroxylated leucocyanidin, whereas those compounds derived from 4′-hydroxylated leucopelargonidin are absent in pigmented rice grains, which implies that activity of F3′H is prominent in pigmented rice grains. Two rice *F3′H* genes belonging to the cytochrome P450 family, *CYP75B3* and *CYP75B4*, have been identified. CYP75B3, which catalyzes the 3′-hydroxylation of the B-ring of flavonoids, was identified by complementation of the *Arabidopsis thaliana transparent testa* mutant 7 (*tt7*), which has a defective allele for F3′H [22]. CYP75B4 was characterized more recently and catalyzes not only the 3′-hydroxylation of flavonoids, but also the 5′-hydroxylation of the 3′-methoxylated flavone chrysoeriol to generate selgin, which is further converted to tricin in a reaction catalyzed by *O*-methyltransferase [19].

Rice grain pigmentation is determined by the functional activities of transcription factors. The *Kala3* gene, which encodes R2R3-Myb, and the *Kala4* gene, which encodes basic helix-loop-helix (bHLH), activate the flavonoid biosynthesis genes, including *CHS*, *DFR*, and *ANS* in black rice, resulting in anthocyanin pigment accumulation in the grain [23,24]. The *Rc* gene encoding bHLH activates *CHS*, *DFR*, and *LAR* in red rice, resulting in proanthocyanidin pigment accumulation in the grain [25]. In white rice, the promoter of *Kala4* has a different structure to that of pigmented rice, and 14 base pairs were deleted within the open reading frame of the *Rc*, which causes an absence of pigment in the grain. Oikawa et al. [24] showed that *CYP75B3* is highly expressed along with other flavonoid pathway genes in pigmented rice grains. This is in accordance with the predominant accumulation of leucocyanidin-derived anthocyanins and proanthocyanidins in pigmented rice grains. However, it is still unclear whether 3′-hydroxylation takes place at the flavanone level or the dihydroflavonol level and why leucopelargonidin derivatives are absent, because of a lack of information about the enzymatic properties of CYP75B3, CYP75B4, and some of the other flavonoid pathway enzymes, and about metabolon formation in the flavonoid pathway in rice.

Aiming to evaluate the roles of *CYP75B3* and *CYP75B4* in flavonoid biosynthesis in rice grain, we isolated the coding regions of these genes from Korean varieties of white, black, and red rice. Consequently, we identified allelic variants of each gene in different varieties. These genes were heterologously expressed in yeast to evaluate their enzyme activities and substrate preferences, and the changes in transcript levels of *CYP75B3* and *CYP75B4* during the development of pigmented and non-pigmented rice grain were analyzed along with other flavonoid pathway genes. In addition, their relative expression levels in other tissues of rice seedlings were also examined.

## 2. Results

### 2.1. Sequence Analysis of CYP75B3s and CYP75B4s

**Figure 2:**
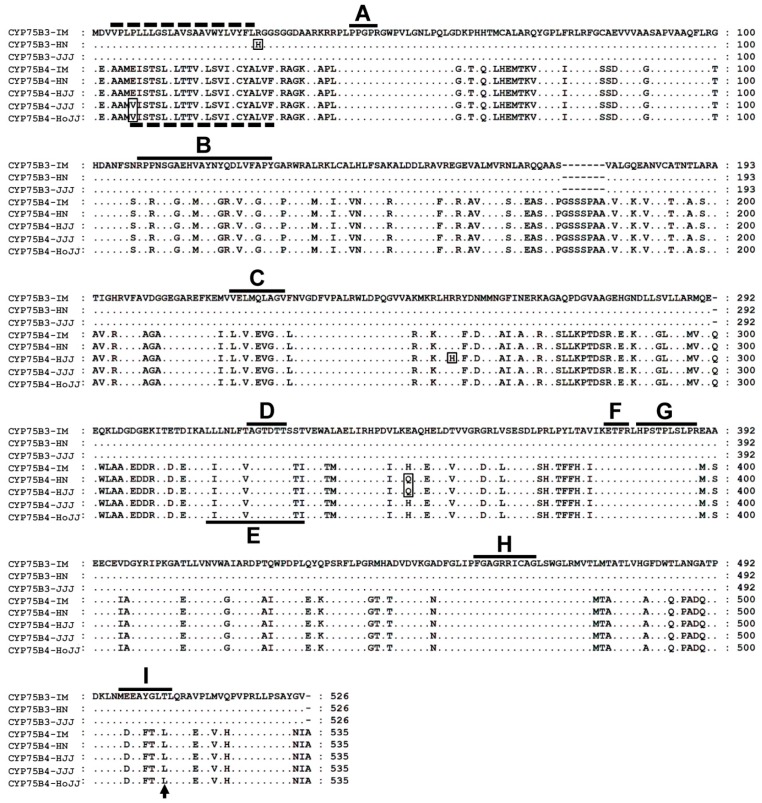
Amino acid sequence alignment of CYP75B3s and CYP75B4s. Deduced amino acid sequences of CYP75B3 and CYP75B4 proteins from the white (IM), black (HN and HJJ), and red rice varieties (JJJ and HoJJ) were compared. Amino acid substitutions were indicated with rectangles. The two dashed lines located in the N-terminal region indicate the predicted membrane spanning anchors of CYP75B3 and CYP75B4 and the solid lines indicate specific conserved regions of cytochrome P450 enzymes. A: hinge region, B: substrate recognition site 1 (SRS1), C: SRS2, D: oxygen binding pocket, E: SRS4, F: ExxR motif, G: SRS5, H: heme biding domain, and I: SRS6. Thr504 of CYP75B3 and Leu512 of CYP75B4 correspond to the previously described functional determinant for F3′H activity, which are indicated by an arrow.

cDNA clones of *CYP75B3* and *CYP75B4* were isolated from the white rice, Iimi (IM), black rice, Heugnam (HN) and Heugjinju (HJJ), and red rice, Jeogjinju (JJJ) and Hongjinju (HoJJ). Three cDNA clones of *CYP75B3* encoded proteins of 526 amino acids (CYP75B3-IM, CYP75B3-HN, and CYP75B3-JJJ) and five cDNA clones of *CYP75B4*-encoded proteins of 535 amino acids (CYP75B4-IM, CYP75B4-HN, CYP75B4-HJJ, CYP75B4-JJJ, and CYP75B4-HoJJ). Amino acid sequence comparison (Figure 2) revealed that CYP75B3-IM and CYP75B3-JJJ are identical to the registered sequence in the public database, but one amino acid was substituted in the CYP75B3-HN sequence at position 27. CYP75B4-IM was identical to the registered sequence in the public database, but an amino acid substitution at position 351 was shared by CYP75B4-HN and CYP75B4-HJJ from black rice and another substitution at positions seven was shared by CYP75B4-JJJ and CYP75B4-HoJJ from red rice. An additional substitution was observed in the CYP75B4-HJJ sequence at position 258. His27 of CYP75B3-HN and Val7 of CYP75B4-JJJ and CYP75B4-HoJJ were located at the N-terminal membrane anchor region; however, the other cytochrome P450-specific conserved regions, such as the oxygen binding pocket, the ExxR motif, and the heme binding domain, were identical among all of the isolated genes, while most of the substrate recognition sites (SRS) were different between CYP75B3s and CYP75B4s, except for SRS5. The Thr504 of each CYP75B3 and the Leu512 of each CYP75B4, known as functional determinants for the specific enzyme activities, were conserved [19,26]. The other substitutions, His258 of CYP75B4-HJJ and Gln351 of CYP75B4-HN and CYP75B4-HJJ, were located out of the functional regions.

### 2.2. Yeast Expression of CYP75Bs and CYP75B4s and Enzyme Assays

**Figure 3:**
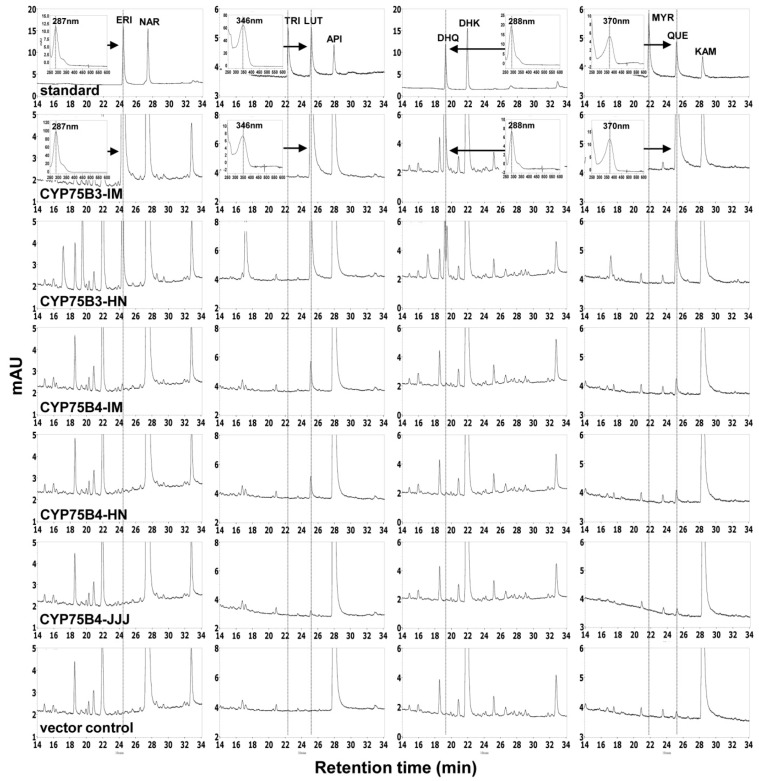
HPLC profiles of yeast transformed with CYP75Bs, CYP75B4s, and vector control. Induced yeast cells were fed with naringenin (NAR), apigenin (API), dihydrokaempferol (DHK), and kaempferol (KAM), respectively, and production of their 3′-hydroxylated (eriodictyol (ERI), luteolin (LUT), dihydroquercetin (DHK), and quercetin (QUE)) and 3′,5′-hydroxylated products (tricetin (TRI) and myricetin (MYR)) were analyzed after 7.5 h incubation. The products were identified according to retention time and UV spectra of authentic standards. The UV spectra of standards are displayed as insets and the corresponding peaks in the chromatograms are indicated by arrows.

We cloned the coding regions of two *CYP75B3*s (*CYP75B3*-IM and *CYP75B3*-HN) and of three *CYP75B4*s (*CYP75B4*-IM, *CYP75B4*-HN and *CYP75B4*-JJJ) into the yeast expression vector pYES-DEST52. *CYP75B3*-JJJ was not cloned, as its sequence is identical to that of *CYP75B3*-IM. All of the constructs, including the empty vector pYES-DEST52, were transformed into yeast strain WAT11. The transformed yeast cultures, induced by galactose, were supplied with apigenin and kaempferol and the racemic mixtures of naringenin and dihydrokaempferol for 7.5 h and the formation of each 3′-hydroxylated product by F3′H activity was analyzed by high-performance liquid chromatography (HPLC) (Figure 3). All five recombinant proteins exhibited F3′H activity in the substrate feeding assay but did not exhibit F3′5′H activity. CYP75B3-IM and CYP75B3-HN metabolized all substrates tested, and CYP75B4-IM, CYP75B4-HN, and CYP75B4-JJJ metabolized naringenin, apigenin, and kaempferol, but did not metabolize dihydrokaempferol. CYP75B3s metabolized much higher levels of substrates compared to CYP75B4s.

To determine kinetic values of the recombinant proteins, we conducted kinetic analyses with microsomal proteins for NADPH-dependent flavonoid 3′-hydroxylation with naringenin, apigenin, dihydrokaempferol, and kaempferol as substrates (Table 1). The kinetic analyses were conducted with only CYP75B3-IM, CYP75B3-HN, and CYP75B4-IM, since CYP75B4-HN and CYP75B4-JJJ showed extremely low activities in the substrate feeding assay. The *K*_m_ values of CYP75B3-IM for naringenin, apigenin, dihydrokaempferol, and kaempferol were measured to be 0.286, 0.072, 2.494, and 0.110 µM, and their *V*_max_ values were 0.214, 0.131, 0.430, and 0.240 μM∙min^−1^·mg^−1^, respectively. The *K*_m_ values of CYP75B3-HN for these flavonoids were 0.108, 0.085, 1.034, and 0.098 µM, and their *V*_max_ values were 0.193, 0.161, 0.281, and 0.270 µM∙min^−1^·mg^−1^, respectively. The catalytic efficiency (*V*_max_/*K*_m_) of CYP75B3-IM for dihydrokaempferol was 4.3-, 10.5-, and 12.6-fold lower than those for naringenin, apigenin, and kaempferol, respectively, and the catalytic efficiency of CYP75B3-HN for dihydrokaempferol was 6.6-, 7.0-, and 10.2-fold lower than those for naringenin, apigenin, and kaempferol, respectively, which indicated that kaempferol is the preferred substrate and dihydrokaempferol is the poorest substrate for CYP75B3-IM and CYP75B3-HN (Table 1). Interestingly, the catalytic efficiencies of CYP75B3-HN for naringenin and dihydrokaempferol were approximately two-fold higher than those of CYP75B3-IM for naringenin and dihydrokaempferol. This discrepancy might be attributed to the single amino acid substitution in the N-terminal region of CYP75B3-HN. Dihydrokaempferol was excluded from the analyses as a substrate in the kinetic analyses of CYP75B4 because it was not metabolized by CYP75B4s in the substrate feeding assay (Figure 3). CYP75B4-IM exhibited, respectively, 0.240 and 2.948 µM of *K*_m_ values and 0.008 and 0.007 µM∙min^−1^·mg^−1^ of *V*_max_ values for apigenin and kaempferol. The catalytic efficiency of CYP75B4-IM for apigenin was 17-fold higher than that for kaempferol, indicating that apigenin is the optimum substrate for F3′H activity of CYP75B4. Naringenin was not converted to eriodictyol in the in vitro assay, but was metabolized in the substrate feeding assay by CYP75B4-IM; therefore, naringenin is the poorest substrate for CYP75B4-IM (Table 1). In comparison with F3′H from other plant species assayed through yeast microsomal expression, overall levels of the *K*m values of CYP75B3s were lower than those of *Fragaria vesca* (4–48 µM) [27] and *Camellia sinensis* F3′Hs (17–44 µM) [28] and comparable to those of *Dahlia variabilis* F3′H (0.5–3.5 µM) [29], whereas the *V*_max_ values of CYP75B3s were lower than those of the *Fragaria vesca* (18–210 µM∙min^−1^·mg^−1^) and *Dahlia variabilis* F3′Hs (7–22 µM∙min^−1^·mg^−1^) but higher than those of the *Camellia sinensis* F3′H (10–49 pM∙min^−1^·mg^−1^).

**Figure 4:**
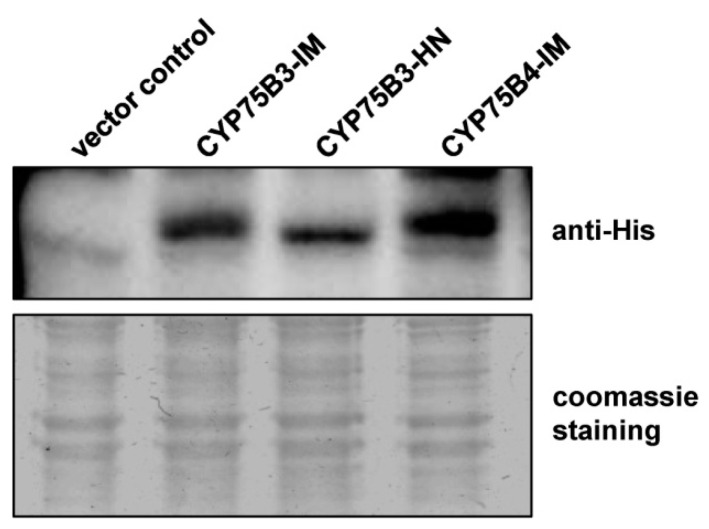
Immunoblot analysis of yeast microsomal proteins containing CYP75B3s and CYP75B4. Equal amounts of microsomal proteins (15 µg) prepared from yeast cells expressing vector control, CYP75B3-IM, CYP75B3-HN, and CYP75B4-IM were subjected to immunoblot analysis with antibody that recognizes the 6xHis tag fused to recombinant proteins (**upper panel**), and were stained with Coomassie Blue R-250 (Biosesang, Seongnam, Korea) (**lower panel**) after SDS-PAGE.

We performed an immunoblot analysis with equal amounts of the microsomal proteins to verify the expression levels of the recombinant proteins in the microsomes (Figure 4). The expression levels of CYP75B3-IM and CYP75B4-IM proteins were comparable but CYP75B3-HN expression levels were approximately two- to three-fold lower than those of the other proteins. This result indicated that the large differences between F3′H activities of CYP75B3s and CYP75B4 did not result from the difference in the amount of recombinant proteins in microsomes. Therefore, it was clear that, overall, F3’H activities of CYP75B3 for various substrates were significantly higher than those of CYP75B4.

### 2.3. Gene Expression Analysis

**Figure 5:**
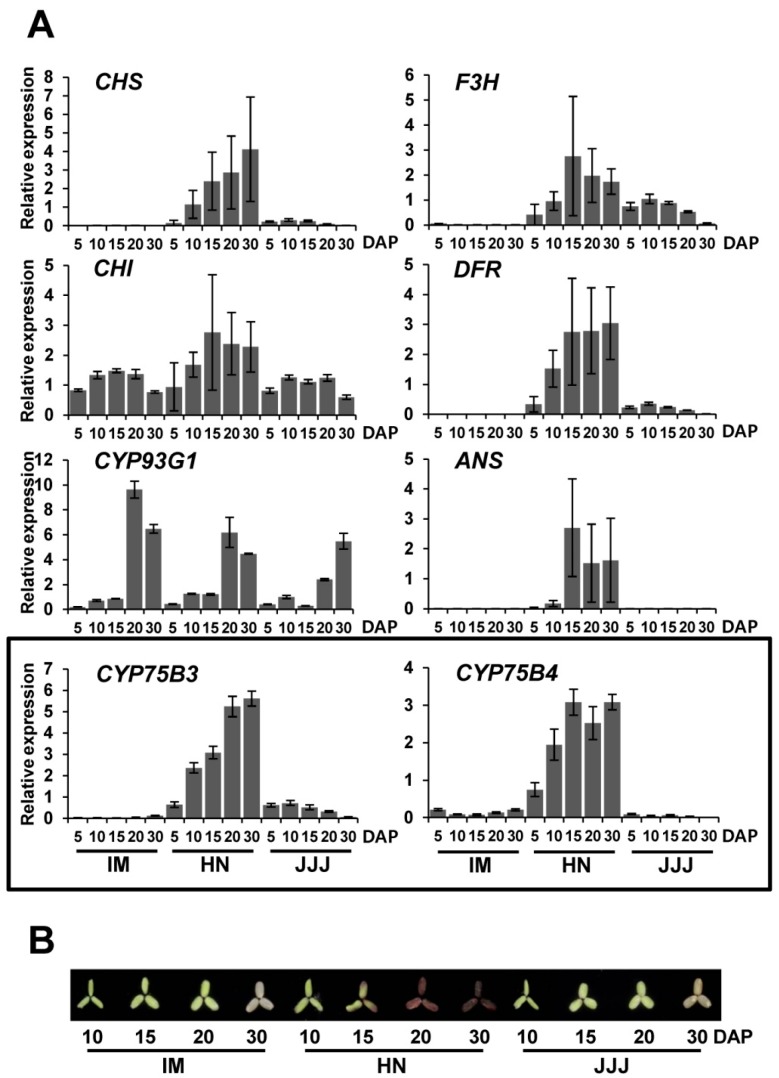
Expression of the flavonoid pathway genes in the seeds of white, black, and red rice varieties, and pigmentation patterns of the seeds during maturation. (**A**) Relative expression patterns of flavonoid pathway genes, including *CYP75B3* and *CYP75B4*, in IM, HN, and JJJ seeds at different time points during maturation (5, 10, 15, 20, and 30 DAP) were analyzed by quantitative PCR (qPCR). *UBQ5* expression was used as an internal reference. The data represent the mean ± SD of three replicates; (**B**) Pigmentation patterns of dehulled IM, HN, and JJJ seeds during maturation.

We compared the expression patterns of *CYP75B3*, *CYP75B4*, and other rice flavonoid pathway genes, including *CHS*, *CHI*, *FNSII* (*CYP93G1*), *F3H*, *DFR*, and *ANS*, in the seeds of non-pigmented and pigmented rice during maturation. IM, HN, and JJJ were used as representative samples of white, black, and red rice varieties, respectively (Figure 5A). All of the genes, except for *CYP93G1*, were expressed predominantly in HN seeds. *CHS*, *DFR*, *CYP75B3*, and *CYP75B4* showed similar expression patterns in the HN seeds, with expression being low on five days after pollination (DAP) and gradually increasing to a maximum by 30 DAP, while the expression levels of *CHI, F3H*, and *ANS* increased to a maximum by 15 DAP, and then decreased over time. *CYP93G1* expression level remained low until 15 DAP, and then rapidly increased to a maximum by 20 DAP. Overall expression levels of these genes in JJJ seeds were much lower than those in the HN grain, and the expression levels of *CHS*, *CHI*, *CYP75B3*, *F3H*, and *DFR* reached a maximum at 10 DAP, and then gradually decreased by 30 DAP. The transcripts of *CHS*, *CYP75B3*, *F3H*, *DFR*, and *ANS* were barely detectable in IM seeds, whereas *CHI*, *CYP93G1*, and *CYP75B4* were expressed at high or detectable levels. On the whole, the expression patterns of *CHI* and *CYP93G1* were distinct among genes examined, which suggests that the expression of these two genes might be minimally affected by the *Kala4* or *Rc* regulatory mechanisms, unlike the other genes. The expression patterns in the HN seeds reflect a highly induced anthocyanin pathway, and the predominant expression of *CYP75B3* and *CYP75B4* represents an abundance of leucocyanidin-derived anthocyanin in black rice grains and substantial quantities of tricin in pigmented rice grains [17], while the relatively low levels of gene expression in JJJ imply that the metabolic flow toward proanthocyanidin and tricin biosynthesis in JJJ seeds is not plentiful compared to HN seeds. The expression of *CYP75B3* in the IM seeds was almost absent, but high levels of *CYP93G1* and low levels of *CYP75B4* expression were observed, which corresponds with tricin being the major flavonoid in white rice grains [18,19]. The anthocyanin pigmentation pattern in the HN seeds during maturation was consistent with the gene expression patterns in the HN seeds, whereas pigmentation was not observed in the JJJ seeds until 30 DAP, which suggests that the relatively low expression levels of most of the genes in the JJJ seeds might be insufficient to accumulate visible levels of pigment in the JJJ seeds until 30 DAP (Figure 5B).

**Figure 6:**
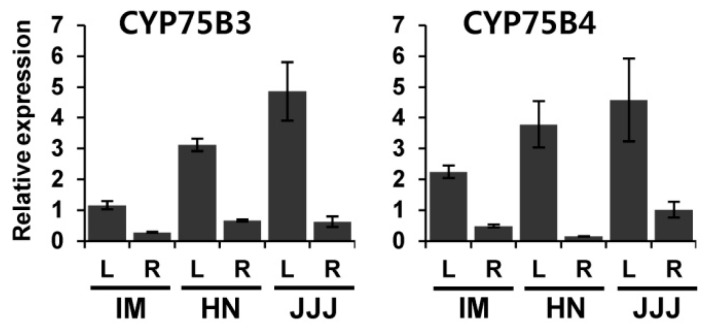
Expression of *CYP75B3* and *CYP75B4* in leaves and roots of white, black, and red rice varieties. Relative expression patterns of *CYP75B3* and *CYP75B4* in leaves (**L**) and roots (**R**) of IM, HN, and JJJ were analyzed by qPCR. *UBQ5* expression was used as an internal reference. The data represent the mean ± SD of three replicates.

The expression patterns of *CYP75B3* and *CYP75B4* in different tissues of seedlings were also examined. Both genes were highly expressed in the leaves compared to the roots, and the expression levels of both genes in the leaves were highest in the JJJ and lowest in the IM. As with the expression in the developing seeds, the expression levels of *CYP75B4* were relatively higher than those of *CYP75B3* in the leaves and roots of IM (Figure 6).

## 3. Discussion

The types and contents of flavonoids in rice grains vary among the different colored varieties and even among varieties of the same color. For instance, the Korean black rice variety, HN, was found to contain 0.6 mg·g^−1^ of cyanidin-3-*O*-glucoside and 0.023 mg·g^−1^ of peonidin-3-*O*-glucoside, while another Korean black rice variety, HJJ, contained 3.02 mg·g^−1^ of cyanidin-3-*O*-glucoside and 0.09 mg·g^−1^ of peonidin-3-*O*-glucoside [13]. In another study, the Italian black rice variety “Artemide”, which is phylogenetically distant from the other black rice varieties HN and HJJ, contained 1 mg·g^−1^ and 0.12 mg·g^−1^ of the respective anthocyanins. Moreover, Artemide contained 0.033 mg·g^−1^ of mavidin-3-glucoside derived from 3′,5′-dimethoxy delphinidin [15]. Between the two Korean black rice varieties included in our study, there was approximately a four- to five-fold difference in the contents of anthocyanins, but the ratios of cyanidin to peonidin were similar (26:1 in HN and 33:1 in HJJ), whereas, in Artemide, the ratio (8:1) differed markedly from those in the Korean varieties. These findings suggest that some allelic variants of the flavonoid pathway genes may be present in the genomes of diverse rice varieties. The majority of known F3′5′Hs among diverse species are members of the CYP75A subfamily. CYP75A11, a single CYP75A member in rice, was known to have non-functional F3′5′H activity [19]. Therefore, the presence of mavidin-3-glucoside in Artemide grain implies that Artemide may have a functional CYP75A11 or an F3′H possessing F3′5′H activity. In our results, the Thr504 and Leu512 residues, which are regarded to be functional determinants for the specific activities of CYP75B3 and CYP75B4, respectively, were conserved in both proteins (Figure 2), suggesting that the functions of these enzymes were not altered to exhibit F3′5′H activity. Correspondingly, we did not detect the 3′5′-hydroxylated products of the flavonoid substrates in the substrate feeding assay (Figure 3) or in the in vitro assay. Interestingly, there was a single amino acid difference between CYP75B3-IM and CYP75B3-HN in the N-terminal membrane anchor region, and CYP75B3-HN exhibited lower *K*_m_ values for naringenin and dihydrokaempferol, resulting in approximately a two-fold increase in catalytic efficiencies for these substrates in comparison to CYP75B3-IM (Table 1), which suggests that the N-terminal region of CYP75B3 plays an additional role in specifying substrates, and the increased preferences for the substrates in the activity of CYP75B3-HN might contribute to the large accumulation of anthocyanins in HN grains.

The kinetic values revealed that CYP75B3s exhibited remarkably lower catalytic efficiencies for dihydrokaempferol than for naringenin, apigenin, and kaempferol (Table 1). Particularly, the catalytic efficiencies of CYP75B3-IM and CYP75B3-HN for dihydrokaempferol were, respectively, 4.3- and 6.7-fold lower than those for naringenin, which suggests that 3′-hydroxylation catalyzed by CYP75B3 may occur at the flavanone level rather than the dihydroflavonol level in rice grains. Therefore, it can be speculated that naringenin is preferentially 3′-hydroxylated to eriodictyol by CYP75B3, and then F3H converts eriodictyol to dihydroquercetin, which is acted upon by DFR and ANS, or DFR and LAR to generate anthocyanin or proanthocyanidin, respectively. This hypothesis is supported by the previous finding that F3H prefers eriodictyol to naringenin [30].

The flavonoid biosynthesis enzymes likely function as a metabolon, which facilitates the direct transfer, or channeling of active sites [31]. Previously, Shih et al. [22] showed a possibility that metabolon formation in rice consists of CHS, F3′H, F3H, DFR, and ANS, which is plausible because extremely low levels of intermediates were found in pigmented rice grains. Therefore, anthocyanin or proanthocyanidin biosynthesis may proceed within the metabolon without releasing intermediates, and CYP75B3 may act prior to F3H in the sequential arrangement of the enzymes composing the metabolon.

In the tricin pathway in rice, CYP93G1 (OsFNSII) converts naringenin to apigenin, and then CYP75B4 converts apigenin to luteolin, which is further metabolized to tricin through the *O*-methyltransferase activity and the chrysoeriol 5′-hydroxylase activity of CYP75B4 [19]. Our results indicated that apigenin is the best substrate and naringenin is the worst substrate for the F3′H activity of CYP75B4 (Figure 3 and Table 1), which matches up with the functional property of CYP75B4 in tricin biosynthesis. According to the current understanding of the rice flavonoid pathway in pigmented rice grains, F3H, CYP93G1, and CYP93G2 (OsF2H: flavanone 2-hydroxylase) compete for naringenin [32,33]. However, the substrate preferences of F3H [30] and CYP75B3 suggest that CYP75B3, CYP93G1, and CYP93G2 may compete for naringenin in pigmented rice grains. In white rice grains, *F3H* and *CYP75B3* were almost not expressed (Figure 5A); thus, only CYP93G1 and CYP93G2 may compete for naringenin. CYP93G2 converts naringenin to 2-hydroxyflavanone, which is further converted to *C*-glycosylflavone by the activities of *C*-glucosyltransferase (OsCGT) and dehydratase (DH) [34]. However, CYP93G2 seems to play a smaller role than CYP93G1 in the competition for naringenin in vivo, because the major flavone in white rice grains is tricin and not *C*-glycosylflavone.

The tricin content in pigmented rice grain is as much as 16 times lower than the total anthocyanin content [17]; however, the expression levels of both *CYP75B3* and *CYP75B4* increased in accordance with anthocyanin accumulation in black rice grains (Figure 5A,B), suggesting that the lower levels of tricin compared to anthocyanin contents in pigmented rice grains might be attributed to relatively low F3′H activity of CYP75B4 compared to that of CYP75B3. It seems that the low levels of tricin in pigmented rice grains did not result from an insufficient supply of apigenin to CYP75B4, because the increase of *CYP93G1* expression during seed maturation in the HN and the JJJ (Figure 5A,B) reflects a sufficient supply of apigenin to CYP75B4 for the tricin biosynthesis. According to a previous investigation, the relative levels of apigenin accumulation in a CYP75B4 knockout mutant rice seedling were significantly higher than those in a wild-type seedling, despite *CYP75B3* being equally expressed in both seedlings [19], indicating that there is a limit to utilizing apigenin as a substrate by CYP75B3, which also underpins the hypothesis that rice F3′H participates in anthocycanin biosynthesis as a member of the metabolon. Therefore, the role of CYP75B3 in luteolin generation in the tricin biosynthesis pathway could be neglected, although the catalytic efficiency of CYP75B3 for apigenin was found to be approximately 53-fold higher compared to CYP75B4 in this study (Table 1).

The expression level of *CHS* is usually very low in white rice grains [24]. Likewise, *CHS* expression in IM developing seeds was barely detectable, as shown in Figure 5A. Nevertheless, substantial quantities of apigenin and tricin were detected in white rice grains [13,18]. Our results suggested that even though the expression levels of *CYP75B4* in the IM seeds and the F3′H activity of CYP75B4 were very low (Figure 5A and Table 1), highly-expressed *CYP93G1* could maximize the apigenin supply in the IM seeds.

Both *CYP75B3* and *CYP75B4* were substantially expressed in the leaves and roots of the IM seedlings (Figure 6). Moreover, the expression levels of *CYP75B4* were relatively higher than *CYP75B3* in these tissues like their expression patterns in the IM developing seeds. From these results, it can be speculated that the increased physiological and environmental risks caused by the lack of flavonoids in non-pigmented rice would be partially attenuated by the increase of tricin resulting from the enhanced level of *CYP75B4* expression.

Our results reported here provide valuable information for metabolic engineering efforts aimed at increasing the abundance of useful flavonoids in rice grain. Furthermore, our findings provide insight into the flavonoid pathway in rice.

## 4. Materials and Methods

### 4.1. Plant Materials

Seeds of the following non-pigmented and pigmented rice (*Oryza sativa* L.) varieties were obtained from the Agricultural Genetic Resources Center at the National Academy of Agricultural Science (Jeonju, Korea): white rice, Ilmi (IM); black rice, Heugnam (HN) and Heugjinju (HJJ); and red rice, Jeogjinju (JJJ) and Hongjinju (HoJJ).

### 4.2. Isolation of CYP75B3 and CYP75B4 cDNAs from Non-Pigmented and Pigmented Rice

Total RNA samples were extracted from seeds of IM, HN, HJJ, JJJ, and HoJJ harvested 20 DAP using Fruit-mate for RNA Purification (Takara, Otsu, Japan) and Plant RNA Purification Reagent (Invitrogen, Carlsbad, CA, USA) according to the manufacturers′ instructions. The first-strand cDNAs were synthesized with amfiRivert cDNA Synthesis Platinum Master Mix (GenDEPOT, Barker, TX, USA). To amplify the coding regions of *CYP75B3* and *CYP75B4* without the stop codon, gene-specific primers were designed based on the two sequences in the public database. PCR was performed in 50-μL reactions containing 1 µL of four-fold-diluted cDNA, 0.4 µM of each primer (Table 2), PrimeSTAR HS DNA polymerase, and 5× reaction buffer (Takara) under the following conditions: 98 °C for 2 min; 30 cycles at 98 °C for 10 s, 60 °C for 15 s, 72 °C for 2 min; and a final extension at 72 °C for 5 min. The amplicons were subcloned into the pENTR-SD/D-TOPO vector (Invitrogen) via directional cloning technology and verified by DNA sequencing.

### 4.3. Yeast Expression of CYP75B3s and CYP75B4s and Substrate Feeding Assay

Each coding regions of the five cDNAs cloned into the entry vector (pENTR-*CYP75B3*-IM, pENTR-*CYP75B3*-HN, pENTR-*CYP75B4*-IM, pENTR-*CYP75B4*-HN, and pENTR-*CYP75B4*-JJJ) were transferred to a yeast expression vector pYES-DEST52 in frame with the V5 and 6×His tag using Gateway™ LR clonase (Invitrogen). The resulting plasmids were transformed into the yeast strain WAT11 in which the endogenous NADPH-cytochrome P450 reductase has been replaced with an *Arabidopsis thaliana* NADPH-cytochrome P450 reductase, *ATR1* [35]. Yeast was also transformed with the empty vector pYES-DEST52 as a control. Yeast transformation was performed as previously described [36]. Transformants were grown in 50 mL of SGI medium (3.4 g·L^−1^ yeast nitrogen base, 5 g·L^−1^ Bacto Casamino Acids, 40 mg·L^−1^ tryptophan, 150 mg·L^−1^ adenine hemisulfate and 20 g·L^−1^ glucose) at 28 °C for 24 h. Cells collected from the SGI medium were transferred into 50 mL of SLI medium (3.4 g·L^−1^ yeast nitrogen base, 5 g·L^−1^ Bacto Casamino Acids, 40 mg·L^−1^ tryptophan, 150 mg·L^−1^ adenine hemisulfate and 20 g·L^−1^ galactose) to induce protein expression and incubated at 20 °C for one day. Induced cells were spun down and resuspended in fresh SLI medium containing 50 μM substrates and the cultures were incubated at 28 °C for 7.5 h. Then, 1 mL aliquots of the cultures were sonicated and extracted with 700 µL of ethyl acetate. After evaporation, the residues dissolved in methanol were subjected to high-performance liquid chromatography (HPLC). Flavonoid substrates and standards used in this study are as follows: (±)-naringenin, (±)-eridodictyol, apigenin, luteolin, tricetin, (±)-dihydrokaempferol, (±)-dihydroquercetin, kaempferol, quercetin, and myricetin.

### 4.4. Microsomal Protein Preparation

Yeast transformants induced in 50 ml of SLI medium were collected by centrifugation (5000× *g*, 5 min, 4 °C) and the pellets were resuspended in 10 mL of TEK (50 mM Tris pH 7.5, 1 mM EDTA, and 100 mM KCl). After centrifugation (5000× *g*, 5 min, 4 °C), collected cells were resuspended in TES (50 mM Tris pH 7.5, 1 mM EDTA, 0.6 M sorbitol, 20 mM β-mercaptoethanol, and 1 mM phenylmethylsulfonyl fluoride) and were disrupted by bead beating using glass beads. NaCl and PEG4000 were added to the microsome-containing supernatants obtained by centrifugation (5000× *g*, 5 min, 4 °C) to final concentrations of 150 mM and 0.1 g·mL^−1^, respectively. After incubation on ice for 15 min, the microsomal fractions were collected by centrifugation (10,000× *g*, 10 min, 4 °C) and resuspended in 200 µL of TEG (50 mM Tris pH 7.5, 1 mM EDTA, and 30% glycerol). The aliquots of the microsomal fractions were shock frozen in liquid nitrogen and then stored at −80 °C until further use. The concentrations of microsomal proteins were determined by the Bradford method [37] with bovine serum albumin as the standard.

### 4.5. Standard Enzyme Assay

Microsomal protein was incubated in 100 µL of total reaction containing 100 mM Tris pH 7.5, substrate, and 1 mM NADPH, or without NADPH (as a negative control), at 30 °C for 10–30 min. The reaction was stopped by the addition of 37.5 µL of stop solution (acetonitrile/concentrated HCl (99/1, *v*/*v*)). After centrifugation, clear supernatant was subjected to HPLC.

### 4.6. HPLC Analysis

HPLC analysis was performed on an LC-20A HPLC system (Shimadzu, Kyoto, Japan) equipped with an Inertsil-ODS3 C18 column (5 μm, 250 mm × 4.6 mm, GL Science, Tokyo, Japan). The mobile phase was composed of water containing 0.1% formic acid (A) and acetonitrile containing 0.1% formic acid (B). The gradient profile was optimized as follows: 0–30 min, linear gradient 5%–55% (*v*/*v*) B; 30–45 min, linear gradient 55%–65% (*v*/*v*) B; 45–50 min, linear gradient 65%–100% (*v*/*v*) B at a flow rate of 1 mL·min^−1^. The temperature of the column compartment was maintained at 40 °C. A diode-array detector was used to detect compounds. The spectra of the compounds were recorded between 210 and 800 nm. The compounds were identified by comparing the retention times and UV spectra with those of the standards.

### 4.7. Immunoblot Analysis

Yeast microsomal proteins (15 µg) were separated by 12% SDS-PAGE and were electrotransferred onto a polyvinylidene fluoride membrane and then placed in blocking buffer (50 mM Tris-Cl pH 7.4, 150 mM NaCl, 0.1% Tween 20, and 5% skim milk) at room temperature for 1 h prior to being probed with antibodies. Anti-penta His antibodies (Qiagen, Valencia, CA, USA), diluted 1/2000 with blocking buffer, were applied at 4 °C for 10 h. After washing the membrane with TBS-T buffer (50 mM Tris-Cl pH 7.4, 150 mM NaCl, and 0.1% Tween 20), HRP-conjugated secondary antibodies (Milipore, Bedford, MA, USA), diluted 1/5000 with blocking buffer, were applied at 4 °C for 2 h. Chemiluminescent signals were detected using ECL Western Blotting Detection Reagents (Amersham, Buckinghamshire, UK) and an ImageQuant™ LAS 4000 system (Fujifilm, Tokyo, Japan).

### 4.8. Gene Expression Analysis

Total RNA samples were prepared from the developing seeds harvested at 5, 10, 15, 20, and 30 DAP, and leaves and roots of two-week-old rice seedlings. The first strand cDNAs were synthesized from 2 µg of total RNA using cDNA EcoDry Premix (Oligo dT_18_) (Clontech, Palo Alto, CA, USA). qPCR was performed in 15-μL reactions containing 3 µL of four-fold-diluted cDNA, AccuPower 2× Greenstar qPCR Master Mix (Bioneer, Daejun, Korea), and 0.3 µM of each primer (Table 2) on a BioRad CFX96 Detection System (Bio-Rad Laboratories, Hercules, CA, USA). To normalize the expression of the target genes, *UBQ5* was used as an internal reference. All PCR reactions were performed under the following conditions: 95 °C for 5 min; 40 cycles at 95 °C for 15 s, and 55 °C for 30 s. The amplification specificity was verified by melting curve analysis (55–95 °C). The data were expressed as the mean value of three replicates.

## 5. Conclusions

The coding regions of *CYP75B3* and *CYP75B4* were isolated from Korean varieties of white, black, and red rice, and their enzymatic properties and expression patterns were analyzed. We found that there were allelic variants of each gene containing one or two amino acids substitutions and that the allelic variant of CYP75B3 from black rice showed enhanced catalytic efficiencies for naringenin and kaempferol compared to the CYP75B3s from white and red rice. The best and worst substrates for CYP75B3 were kaempferol and dihydrokaempferol, respectively, and for CYP75B4 were apigenin and naringenin, respectively, but CYP75B4 could not utilize dihydrokaempferol. Overall, the F3′H activities of CYP75B3 for the substrates were much higher than those of CYP75B4. Gene expression analysis revealed that *CYP75B3*, *CYP75B4*, and most flavonoid pathway genes evaluated were expressed predominantly in the developing seeds of black rice and not in those of white and red rice, which is consistent with the pigmentation patterns of the seeds during maturation. Furthermore, these two genes were highly expressed in the leaves and weakly expressed in the roots. In particular, *CYP75B4* expression was relatively higher than *CYP75B3* expression in white rice. These results provide valuable information for better understanding of rice flavonoid pathway.

## Figures and Tables

**Table 1:** Kinetic parameters for 3′-hydroxylation of CYP75B3s- and CYP75B4-containing yeast microsomal proteins.

OsF3′Hs		Substrates			
		NAR	API	DHK	KAM
CYP75B3-IM (CYP75B3-JJJ)	*K*m (µM)	0.286 ± 0.015	0.072 ± 0.011	2.494 ± 0.350	0.110 ± 0.008
	*V*_max_ (μM∙min^−1^·mg^−1^)	0.214 ± 0.015	0.131 ± 0.011	0.430 ± 0.05	0.240 ± 0.014
	*V*_max_/*K*_m_ (1∙min^−1^·mg^−1^)	0.746 ± 0.067	1.809 ± 0.168	0.173 ± 0.018	2.178 ± 0.282
CYP75B3-HN	*K*_m_ (μM)	0.108 ± 0.009	0.085 ± 0.015	1.034 ± 0.107	0.098 ± 0.013
	*V*_max_ (μM∙min^−1^·mg^−1^)	0.193 ± 0.029	0.161 ± 0.008	0.281 ± 0.023	0.270 ± 0.021
	*V*_max_/*K*_m_ (1∙min^−1^·mg^−1^)	1.796 ± 0.240	1.887 ± 0.156	0.271 ± 0.034	2.755 ± 0.323
CYP75B4-IM	*K*_m_ (μM)	ND	0.240 ± 0.042	*	2.948 ± 0.330
	*V*_max_ (μM∙min^−1^·mg^−1^)	ND	0.008 ± 0.001	*	0.007 ± 0.001
	*V*_max_/*K*_m_ (1∙min^−1^·mg^−1^)	ND	0.034 ± 0.003	*	0.002 ± 0.001

ND = Not detectable; * = Not investigated. The data represent the mean ± SD of three independent measurements. NAR, naringenin; API, apigenin; DHK, dihydrokaempferol; KAM, kaempferol.

**Table 2:** List of primers used for qPCR and cloning of cDNAs.

Target	Locus ID	Forward (5′ to 3′)	Reverse (5′ to 3′)
qPCR			
*CYP75B3*	Os10g0320100	ACGGATTCATCAACGAAAGG	AGCAGCACGCTTAGAAGGTC
*CYP75B4*	Os10g0317900	TCTCCCATCCGCTTACAATA	ACCAATCTACCAACATACAACAA
*CHS*	Os11g0530600	GGGCTCATCTCGAAGAACAT *	CCTCATCCTCTCCTTGTCCA *
*CHI*	Os03g0819600	AATCGAGCTGCGAATTAACC *	CGCGATTTCTCCTTTCCTTT *
*FNSII*	Os04g0101400	GATTGGCAGTGCATGGACA	TTGTATTACGGTGCGTTACAGG
*F3H*	Os04g0662600	AGCACAGAAGCCCAAGTCTC *	CTTCGATTTTCGACGGAAGA *
*DFR*	Os01g0633500	GCGAGAAGGAACCGATACTG *	TCCAAATCTCGCATTGTGAA *
*ANS*	Os01g0372500	GCATCGAACGGAATGAGAAC *	TTCGCTTCCGTTGAACATTA *
*UBQ5*	Os01g0328400	GAAGTAAGGAAGGAGGAGGA *	AAGGTGTTCAGTTCCAAGG *
Cloning			
*CYP75B3*		CACCATGGACGTTGTGCCTCTCCCGC	TACTCCATAAGCCGATGGAAGCAGC
*CYP75B4*		CACCATGGAGGTCGCCGCCATGG	TGCAATATTGTAAGCGGATGGGAGAAG

* = Identical sequence to a primer used in a previous study [38].
